# Mycoflora and Ochratoxin A Control in Wheat Grain Using Natural Extracts Obtained from Wine Industry By-Products

**DOI:** 10.3390/ijms13044949

**Published:** 2012-04-19

**Authors:** Ersilia Alexa, Mariana-Atena Poiana, Renata-Maria Sumalan

**Affiliations:** 1 Faculty of Food Processing Technology, Banat’s University of Agricultural Sciences and Veterinary Medicine, Calea Aradului 119, RO 300645, Timisoara, Romania; E-Mail: alexa.ersilia@yahoo.ro; 2 Faculty of Horticulture and Forestry, Banat’s University of Agricultural Sciences and Veterinary Medicine, Calea Aradului 119, RO 300645, Timisoara, Romania; E-Mail: srenata_maria@yahoo.com

**Keywords:** ochratoxin A, butylated hydroxytoluene (BHT), grape seed extract, grape pomace extract, fungus genera

## Abstract

The aim of this study was to evaluate the effect of some natural extracts obtained from grape pomace (GPE) and grape seeds (GSE) as compared to a synthetic food, antioxidant-butylated hydroxytoluene (BHT), in order to control fungal population and ochratoxin A (OTA) production in naturally contaminated wheat. The results showed that the addition of these extracts was efficient with OTA content decreasing. On treatment with these extracts the loss of OTA content after 14 days was in the range 7.8–28.3% relative to the control sample, but increased up to 26.48–37% after 28 days. The highest loss in OTA content was recorded for treatment with GPE at the 500 ppm level. Regarding the fungal development, the obtained results show that the total fungal populations were significantly reduced by using natural extracts. The most efficient extract was GPE. Both BHT and GPE inhibit the growth of *Penicillium verrrucosum*, for all doses, but less with *Aspergillus* genera. GPE affects the growth of other types of moulds such as *Rhizopus microsporus*, *Fusarium graminearum*, *Alternaria infectoria* and *Cladosporium herbarum*. Our data suggested that GPE and GSE are able to provide fungicidal and fungistatic protection and to control the OTA accumulation in wheat, at least in a similar manner to BHT.

## 1. Introduction

Mycotoxins are toxic chemical products formed as secondary metabolites by a few fungal species that colonize crops and contaminate them with toxins in the field or after harvest [1]. They are produced during growth and multiplication of fungus when micro ecological conditions are favorable [2]. The most important groups of mycotoxins that occur often in cereals destined for food and feed consumption are: aflatoxins, ochratoxin A, trichothecenes (deoxynivalenol, nivalenol), zearalenone and fumonisins. Ochratoxin A (OTA), a toxin produced by *Aspergillus ochraceus*, *Aspergillus carbonarius* and *Penicillium verrucosum*, is one of the most abundant food-contaminating mycotoxins in the world, that occurs in vegetal products especially in cereals. OTA has been considered as a possible cause of the human disease known as Balkan Endemic Nephropathy (BEN). Also, it is a strong teratogen and its primary harmful effect is general cytotoxicity [3–5]. Prevention of fungal infection during plant growth, harvest, storage and distribution and the measures that must be taken for decontamination represent a current issue for the European Commission [6]. Storage in adequate conditions (moisture, temperature and insect control) and the addition of antifungal agents may diminish fungal growth but can not detoxify contaminated samples. The main techniques to reduce contamination of cereals by mycotoxins are based on physical [7–9], chemical [10–13], microbiological [14] and biotechnological methods [15]. Substances which do not directly interact with mycotoxins, *i.e.*, antioxidant agents and immunostimulatory agents, may be very efficient in reducing the toxicity of mycotoxins [16–18]. Substances with preservative action are often added to moist cereals, especially those for animal feed [18]. Propionic, acetic and formic acid, butylated hydroxianisole (BHA), butylated hydroxytoluene (BHT) and propyl paraben (PP) were applied singly or in combination to assess their effectiveness in preventing the growth of moulds [19]. These compounds control the growth of *Aspergillus*, *Penicillium* and *Fusarium* populations and aflatoxin and fumonisin synthesis [20–24]. Also, BHA and PP inhibit deoxynivalenol and nivalenol production in wheat grain [25–27]. The synthetic phenolic antioxidants (e.g., BHT, BHA) are preferred to fungicides because of their protective effect on health. These compounds added to human and animal food are able to increase the life time of organisms and decrease the incidence of cancer caused by chemical compounds. In the last few years some alternatives to synthetic compounds used in prevention of mycotoxins accumulation have been developed. Natural antioxidants such as resveratrol have been demonstrated to have a particularly wide spectrum on fungal growth and mycotoxin production [20,25]. The effect of synthetic resveratrol on fumonisin and zearaleone production in maize showed a reduction in zearalenone production by *Fusarium graminearum* and inhibition of OTA on natural wheat grain [25,28]. Resveratrol is able to inhibit OTA production for *Penicillium verrucosum* and *Aspergillus westerdijkiae* in naturally contaminated wheat grain and is more effective in fungus control than essential oils [29]. The natural source of resveratrol is grape pomace. Grape pomace represents a valuable low-cost raw material for the extraction of value-added compounds such as polyphenols especially anthocyanins and procyanidins with potential as food additives or nutraceuticals [30–32]. Grape pomace consists of skin, seeds and the pulp remaining from the production of wine. The phenolic compounds in these extracts are responsible for their antioxidant activity and have been reported to possess biological properties such as anti-carcinogenic, anti-mutagenic, anti-inflammatory and antimicrobial properties [33–35]. In this regard, grape pomace has become an ideal candidate as a cost-effective product with natural and high value-added polyphenolic phytochemicals [36]. Antioxidant characteristics of grape seed and grape pomace extracts were evaluated as well as their ability to control the mould growth and mycotoxin production in wheat [28]. Resveratrol, which is found in grape skins, has been proven to possess many functions in modulating physiological and pathological reactions of the body, such as anti-cancer, anti-mutagenesis, and cardioprotection [32].

The effect of synthetic trans-resveratrol and natural extracts obtained from wine industry by-products on *Fusarium* species and mycotoxins production was studied by Marin *et al.* [28,37]. No differences were found when using synthetic trans-resveratrol or natural extracts, suggesting the wine industry by-products are a cheaper source of resveratrol than the synthetic one.

The goal of this research was to investigate the effect of two natural extracts obtained from wine industry by-products compared to a synthetic antioxidant-butylated hydroxytoluene (BHT) in order to control fungal population and OTA production in naturally contaminated wheat grain.

## 2. Results and Discussion

The present study was carried out directly in naturally contaminated wheat. For this purpose, a wheat sample (2000 g) was chemically sterilized so that opportunistic mycoflora would become inactivated. The wheat samples (200 g) separately treated with different concentrations of GPE, GSE and BHT (500, 1000, 2500 ppm) were kept in storage conditions (temperature 20 °C, a_w_ = 0.85) and analyzed after 7, 14, 21 and 28 days. The different antioxidant levels were chosen in agreement with previous studies that have proved that the inhibition of fungus and mycotoxins production increased with the dose used for treatment [28].

The fungal population and level of OTA contamination have been analyzed. To provide a clear view on the changes occurring for the investigated parameters as a result of different types of antioxidant in wheat grain samples, the obtained data were processed by ANOVA one-way test. Based on information obtained by statistical processing, the significance of changes in mycoflora and ochratoxin A content, as response to extracts type and dose can be ascertained.

### 2.1. Antioxidant Properties of Natural Extracts

Antioxidant characteristics of natural extracts (GPE and GSE) and BHT used in this study are reported in Table 1.

Grape skins and seeds are rich sources of health-promoting polyphenols. They contain flavonoids such as catechin, epicatechin, procyanidins and anthocyanins. They also contain phenolic acids that include gallic acid and ellagic acid and stilbenes such as resveratrol [38]. For the grape seeds, the two most abundant phenolic compounds are catechin and epicatechin. Ellagic acid, resveratrol, myricetin, quercetin, and kaempferol were found in the skins and gallic acid was found as one of the phenolic compounds present in grape seeds [39].

On the basis of registered data it can be observed that BHT had the maximal FRAP value (1328.14 μmol Fe^2+^/g) followed by GSE (1042.38 μmol Fe^2+^/g) and GPE (804.17 μmol Fe^2+^/g).

Total phenolics content of GSE and GPE was in agreement with data reported previously in other studies. Many researchers have reported variable phenolic content in GSE ranging from 98.75 mg/g as gallic acid equivalent (in extract obtained from grape seeds of the blue variety when they were extracted with 80% ethanol) to 667.87 mg/g when the extracting medium was an acetone:water:acetic acid mixture (90:9.5:0.5 v/v) [40]. The quantity of total phenolic substances from GSE was higher than for GPE. These results are consistent with the data reported previously by Negro *et al.* [41]. According to Pastrana-Bonilla *et al.* [39], the total phenolics in grape parts were, on average, five times more concentrated in the seeds than in the skin and 80 times more than in the pulp.

### 2.2. The Impact of Treatment with Natural Extracts and BHT on OTA Accumulation

**Figure 1:**
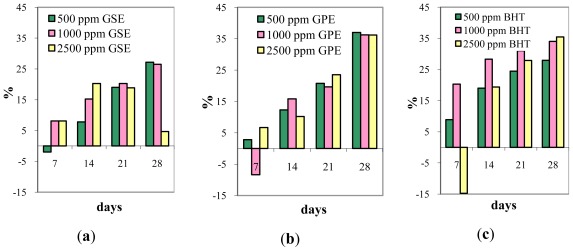
The decline registered in OTA content on treatment with natural extracts (**a)** GSE; (**b**) GPE; (**c**) BHT.

Presented in Table 2 are the changes recorded in OTA content of wheat grain samples during storage showing the effect of treatment with natural extracts and BHT. Also, Figure 1 provides information on the OTA decrease registered in response to treatments with natural extracts or BHT during the storage time relative to the control sample. The initial concentration in the control sample of OTA was 12.93 ppb. After treatment with natural and synthetic antioxidants registered changes in OTA content were in the range 9.00–15.09 ppb, depending on the antioxidant nature, dose and storage time. Previous studies regarding the use of synthetic antioxidants in control of mycotoxins synthesis during storage showed that BHT and BHA, alone or in combination with another antioxidants, are effective in controlling both growth and toxin production on maize and wheat grain under different experimental conditions (concentration, water activity and temperature) [23].

In terms of treatment with BHT and the effect on the OTA accumulation, our results showed that after 28 days from starting treatment, OTA concentration was between 9.12–10.17 ppb. In this time, OTA content increased from 12.93 to 14.12 ppb in the control sample. The results revealed that by increasing the BHT dose from 500 to 2500 ppm the OTA concentration in wheat samples is not largely influenced. After 28 days of starting treatment, BHT addition at the 2500 ppm level induced a decrease in OTA content from 12.93 to 9.12 ppb. Also, BHT at the 1000 ppm level induced a similar decrease of OTA content after the same period. These results demonstrate that the use of high concentrations of BHT (2500 ppm) similar to those suggested by the producing companies (0.2–0.25%), are not justifiable. The results presented in Figure 1 highlight the fact that, during the investigated period, the losses registered in OTA content increased compared to the control sample, except over the first 7 days of storage.

After 14, respectively 21 days from starting treatment, it can be observed that the efficiency of BHT treatment quantified by the losses recorded in OTA content, were higher at the 1000 ppm level (28%, respectively 31%) than those recorded for BHT at the 500 ppm level (19%, respectively 24%) and for the 2500 ppm level (19.36%, respectively 27.87%). After 28 days from starting the experiment, BHT treatment (1000–2500 ppm) induced decreases in OTA content of up to 34–35% relative to the control.

The results presented in Table 2 showed that the treatment with natural extracts (GPE and GSE) were efficient in decreasing OTA accumulation, having at least a similar effect to BHT.

The addition of GSE at the 500 ppm level induced a slow increase of OTA content after seven days, followed by a decrease of OTA concentration after 28 days from starting treatment. By increasing the GSE dose to1000 ppm a moderate decrease of OTA content was induced (12.08 ppb after 7 days, respectively 10.38 ppb after 28 days). The results are in accordance with the study of Fanelli *et al.* [28] which found that resveratrol isolated from grapes and added to wheat and corn seeds could lead to a sharp reduction of OTA.

The treatment with GPE also led to inhibition of OTA synthesis compared to the control sample. GPE at the 500 ppm level and induced a decrease of OTA concentration from 12.93 ppb to 8.89 ppb after 28 days from starting treatment. Previous researches proved that resveratrol extracted from grape was able to completely inhibit OTA production at the 500 ppm level and was more effective than essential oils in controlling OTA synthesis [29]. For treatment with GPE in high concentration (1000 and 2500 ppm) the effects on OTA concentration were similar suggesting no advantage in using a higher dose. Comparable results were also obtained by Reynoso *et al.* [42] when synthetic antioxidants were used in *Fusarium* species control. From Figure 1 it can be seen that after 14 days, the treatments with GSE and GPE induced losses in OTA content in the range 8–28% relative to the control sample. After 28 days, the recorded losses were in the range 26–37% relative to the control sample.

The highest decrease in OTA content was obtained for GPE treatment at the 500 ppm level. Our results are in agreement with those reported by Aldred *et al.* [29] concerning the effect of resveratrol (at 200 ppm level) on OTA production by *Penicillium verrucosum* in stored wheat grain for 28 days, at 25 °C, when the losses registered in OTA content were between 27.12–71.42% relative to the control, depending on water activity. The registered values also prove that, there is no major difference concerning OTA control when using 500–2500 ppm range for natural extracts, which confirms that the inhibition of OTA production is not proportional to the dose of antioxidant agent used for treatment [28]. Some stimulation of OTA production was observed with 500 ppm GSE, 1000 ppm GPE and 2500 ppm BHT, after seven days of treatment which could indicate that, as a response to antioxidants stress, the fungus species, as a survival mechanism, produces more mycotoxins [42]. After 14 days the OTA accumulation decreased compared to the control sample, therefore proving the efficacy of natural and synthetic extracts with antioxidant capacity on OTA development in wheat grain.

The results showed that after 28 days from starting treatment the most efficient in decreasing OTA was GPE followed by BHT and GSE. From Table 1, it can be seen that GPE does not have the highest polyphenol content, *i.e.*, antioxidant capacity. We advance the idea that antifungal activity of extracts could be determined by the type of polyphenolic compounds present. Literature studies indicate that resveratrol, which has proved to be an effective agent to control OTA accumulation in cereals, is found in large amounts in grape skin (0.2 mg/100 g) [30]. GPE was obtained from the whole pomace and, probably contains a higher amount of resveratrol compared with GSE.

More studies are needed in order to prove the mechanisms involved in OTA synthesis inhibition on treatment with natural extracts obtained from wine industry by-products.

From statistical analysis using ANOVA one-way could be observed, that addition of GSE (500 ppm) and GPE (500 ppm, 2500 ppm) to the wheat grain samples induced non-significant modifications (*P* > 0.1) in OTA content after 7 days from starting treatment. After 14 days from starting treatment statistical significant differences in OTA content were recorded: significant (*P* < 0.05) for GSE and highly significant (*P* < 0.01) for GPE at the 500 and 1000 ppm level, but non-significant changes (*P* > 0.1) at 2500 ppm. After 28 days from starting treatment, for all samples, highly significant (*P* < 0.01) and extremely significant (*P* < 0.001) differences were recorded. The BHT treatment induced significant statistical differences in OTA content (*P* < 0.05), after seven days of treatment and highly significant (*P* < 0.01) after 14–28 days, except for the sample treated with 2500 ppm dose, when after 28 days the difference relative to the control was extremely significant (*P* < 0.001).

### 2.3. Impact of Grape Extracts and BHT Treatment on Natural Mycoflora

**Figure 2:**
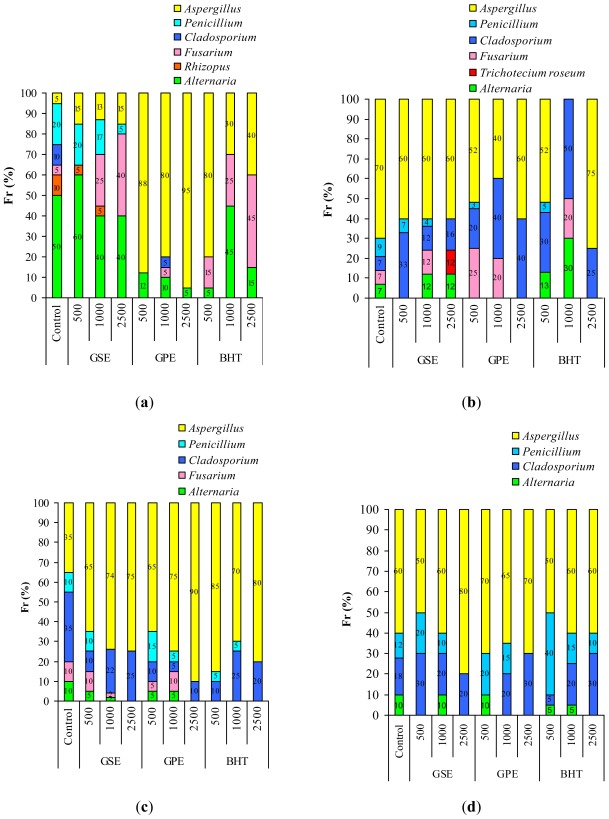
The impact of treatment with GSE, GPE and BHT on Fr of fungus genera during incubation period (**a**) after 7 days; (**b**) after 14 days; (**c**) after 21 days; (**d**) after 28 days.

**Figure 3:**
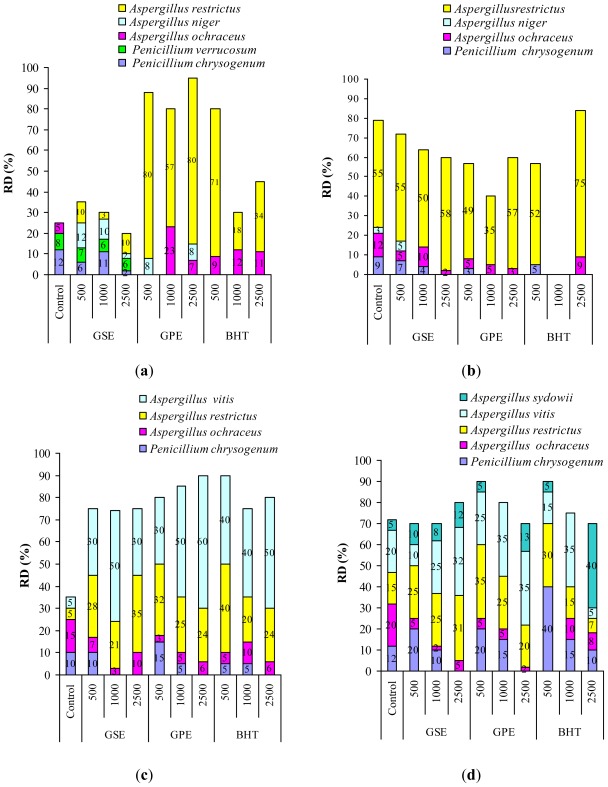
The impact of treatment with GSE, GPE and BHT on RD of fungus species of OTA-producers genera (**a**) after 7 days; (**b**) after 14 days; (**c**) after 21 days; (**d**) after 28 days.

**Figure 4:**
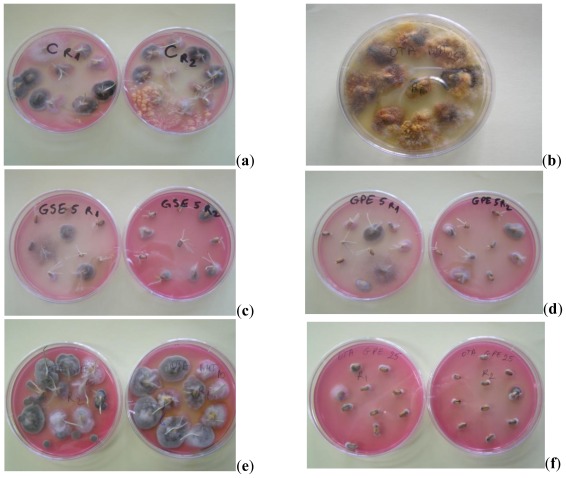
Images of the wheat grain contamination with different treatments applied (**a**) high level of contamination in control sample; (**b**) high level of contamination with *Eurotium amstelodami* (teleomorph of *Aspergillus vitis*) on DG 18 medium; (**c**) sample treated with 500 ppm GSE after 7 days; (**d**) sample treated with 500 ppm GPE after 7 days; (**e**) high level of *Cladosporium* and *Alternaria* in sample treated with 1000 ppm BHT after 14 days; (**f**) sample treated with 2500 ppm BHT after 21 days.

The impact of natural extracts and BHT treatment on the indigenous mycobiota of wheat grain was estimated on the basis of the seed contamination index-SCI(%), the isolation frequency of genera-Fr(%) and relative density RD (%) of OTA-producers of fungus calculated according to Gonzalez [43]. Data from Table 3 express the variation of SCI registered relative to time and applied treatments. presented in Figure 2 is the Fr of fungus genera during incubation period and in Figure 3, RD values of fungal species from OTA-producers genera for the same period. Presented in Figure 4 are the images of the contaminated wheat seeds under different applied treatments. The results reveal that mycoflora behaved differently over time in relation to extract type and dose. The initial, SCI value of the control was 83.3%, and after seven days, small differences were recorded between the SCI of samples treated with natural extracts, BHT and control. The SCI varies in the range 76.67–96.66%, the minimum value was recorded for the sample with 2500 ppm BHT and the maximum for sample with 2500 ppm GPE. The antifungal effect of BHT was proved also by Nesci *et al.* [21]. The highest concentration of GPE (2500 ppm) led to an increase of SCI value which can be explained by the fact that as a response to antioxidants stress, the fungus species are produced [44]. After 14 days of treatment, the total populations of fungi were reduced by the presence of natural extracts and BHT for most of the samples. According to results obtained after data processing by the ANOVA one-way test, these losses are highly significant. Only for the treatment with BHT (1000 ppm), does SCI exceed the control sample, due to high contamination with *Cladosporium* and *Alternaria*, as seen in Figure 2b. The smallest value of SCI was determined for GPE at a level of 1000 ppm. After 21 days the differences between samples treated with GPE (2500 ppm), BHT (1000 ppm) and GSE (500, 100 and 2500 ppm) were extremely significant. At the end of the investigated period, the results were identical in terms of SCI value that show formation of the fungus population balance in accordance with maintaining a constant water activities value [8]. The comparative increase in load mould for samples treated with GPE, GSE and BHT correlates with the results obtained in the first experiment concerning the OTA inhibition.

Regarding frequency of mould genera occurrence we found that the highest value of Fr was recorded for *Alternaria* genus, Figure 2a. After 14 days, there are changes of FR value in terms of mould types, Figure 2b. Thus, decreasing FR values recorded for *Alternaria* genus (*Alternaria alternata* and *Alternaria infectoria*), correlated with doses of GPE, GSE and BHT, while *Rhizopus* completely disappears in samples treated with GSE at the 2500 ppm level.

Development of *Aspergillus* genera in the wheat samples, Figure 2(c,d) can be explained by growth stimulation of species in low humidity conditions, not exceeding 0.85 value [8,19,20]. Figure 3 presents the effect of natural extracts and BHT on OTA-producers. OTA is synthesized especially by *Penicillium verrucosum* and *Aspergillus ochraceus*, the quantity depending on the level of water activity [44,45]. The resulted presented in Figure 3a revealed that after seven days, GSE has no effect on *Penicillium verrucosum*, with RD values recorded of 7%, 6% and 6%. The OTA content for the sample treated with GSE at the 500 ppm level exceeded the control after seven days (Table 2) due to the presence of *Penicillium verrucosum*. This fungus is very active at a_w_ value over 0.8 and the RD value recorder does not correlate with the OTA amount released. From this point of view, our results are in agreement with those obtained by Elmholt and Rasmussen [45].

GPE and BHT inhibited the growth of both ochratoxigenic moulds *Penicillium verrucosum* and *Aspergillus ochraceus*. The efficiency of treatment with GPE was observed after 14 days when the RD value of *Aspergillus ochraceus* is decreased more, compared to the value after 7 days, Figure 3(b–d). In this study GPE affected the growth of other types of mould such as *Rhizopus microsporus*, *Fusarium graminearum*, *Alternaria infectoria* and *Cladosporium herbarum*.

Treatment with BHT inhibits the growth of many fungi, the most obvious effect was found with 1000 ppm BHT where the genus *Aspergillus* is entirely missing.

Overall, decreasing values of Fr were found for *Alternaria* and *Fusarium* but high values of Fr for *Cladosporium*, *Fusarium graminearum* and *Aspergillus restrictus* which is a non mycotoxin producer [46]. Constant conditions in terms of a_w_ value (0.85) during the experiment determined after 28 days showed the occurrence of *Aspergillus vitis*, with its teleomorph *Eurotium amstelodami* and *Aspergillus sydowii*, but neither are responsible for OTA production [46].

The presence of *Aspergillus ochraceus* after 28 days of treatment in most samples can be explained by reaching the tolerance of this species to the action of antioxidants.

It can be seen that the natural extracts have an antifungal effect that is immediately observed and over time the effect is micostatic for fungus ochratoxin producers. Competition between *Aspergillus ochraceus* (*Aspergillus westerdijkiae*) and other species has been shown to have a marked influence on OTA production [47]. Growth of mycotoxigenic species were sometimes inhibited by the presence of other competing fungi, in our case by the *Cladosporium*.

The decline registered in OTA content was at its maximum in the sample treated with BHT at 1000 ppm level (Figure 1) caused by OTA producers of the genera *Aspergillus* and *Penicillium* absent after 14 and 28 days, Table 2. Instead, for this sample it is worth mentioning the large amount of *Cladosporium* species (50%), which determines the growth of SCI over the control value. Both control and treated samples were free from *Aspergillus parasiticus* or *Aspergillus flavus*.

## 3. Experimental Section

### 3.1. Material

Wheat samples. In this work was used naturally contaminated wheat grain (Lovrin 34 variety) harvested in 2010 in the western part of Romania. Wheat samples were taken from farms with inadequate drying and storage conditions. The main physico-chemical characteristics of wheat grain at the starting point of this study were: humidity 13.2%, protein 9.4%, gluten index 9%, Zelleny index 13.

Chemical reagent and microbiological medium. BHT was obtained from Sigma Chemical. Commercial ELISA kits for mycotoxins identification were purchased from R-Biopharm: Kit Elisa-Fast Ochratoxin A lot 12101. The ELISA method validation was carried on a reference certificated material of 64.5 ± 4.9 ppb produced by R-Biofarm P64/OC 852. For mycrobiota evaluation as medium DRBC (dichloran rose bengal chloramphenicol agar) and DG18 (dichloran 18% glycerol agar) were used, produced by Sigma. In addition, AFPA (*Aspergillus flavus* and *parasiticus* agar) medium was used for confirmation of *Aspergillus parasiticus* and *Aspergillus flavus.*

### 3.2. Processing of GSE and GPE

Pressed grape pomace obtained from the Cabernet sauvignon (*Vitis vinifera*) wine production was taken from Recas winery (western part of Romania, vintage 2010). Grape pomace was divided into two parts. One part was processed as whole pomace in order to obtain grape pomace extract (GPE). From the other part, grape seeds were manually removed from the skin and pulp and were used to obtain grape seed extract (GSE). Both grape pomace and separated seeds were dried at 60 °C for 24 h in a drying oven (Binder, Germany) then ground using a grinder (Grindomix Retsch GM 2000). The ground dried seeds were delipidized in a Soxhlet apparatus (Velp, Italy) using hexane, prior to extraction. With regard to phenolic compounds extraction from grape pomace, previous results on this topic were taken into account reported by Spigno and Faveri [48]. Thus, 50 g of grape pomace powder, respectively delipidized seeds powder was mixed in 70% (v/v) ethanol (1000 mL) and kept in a shaking incubator at 25 °C for 48 h and filtered under vacuum then centrifuged (4500 rpm, 15 min). The obtained supernatants were evaporated to 100 mL under reduced pressure at 50 °C using a Rotary evaporator (Heidolph Laborota 4000). The ethanolic extracts were freeze-dried using a lyophilizer (Ilshin Lab Co., Ltd.). The powdered extracts (GPE and GSE) were kept frozen (−18 °C) until further use.

### 3.3. Samples Treatments

A wheat sample (2000 g) was chemically sterilized so that opportunistic mycoflora were inactivated. The sterilization was done with dilute hypochlorite 1:10 (v/v), stirring occasionally for 2 min then draining the solution and rinsing twice with sterile distillate water. Flasks were shaken and equilibrated for 48 h at 4 °C. The wheat sample (200 g) was spiked separately with BHT and natural extracts (GSE and GPE) at three different levels (500, 1000, 2500 ppm).

The natural extracts and BHT were dissolved in 1 mL ethanol 96% (v/v) before adding to the grain. Control samples without antioxidant were also placed under identical conditions. All of the experiments were performed in triplicate. The control sample (200 g) was treated also with 1 mL ethanol. Distilled water was added to obtain the required water activity level (a_w_ = 0.995). The amount of water was calculated from the moisture adsorption curve of wheat grain. To enable ethanol evaporation, the flasks were held at 25 °C for 2 h and periodically mixed. Samples were incubated at 25 °C for 28 days and a_w_ of wheat grain samples was kept at 0.85 by weighing and spraying with sterile water. After 7, 14, 21 and 28 days samples from each experiment were taken in order to assess mycotoxins.

A second experiment was carried out of the seeds for mycoflora analysis using 10 g from each sample treated with GPE, GSE and BHT with the same doses (500, 1000 and 2500 ppm). Grain mycroflora was evaluated initiallly (control), and after 7, 14, 21 and 28 days from starting treatment. During this period the samples were kept in an incubator at 25 ± 2 °C.

### 3.4. Total Phenols Assay

The total phenolic content of grape pomace and seeds extracts was quantified according to the method of Singleton *et al.* [49] with some modifications. A calibration curve using gallic acid was prepared and the absorbance of the standards and samples measured at 750 nm using a UV-VIS spectrophotometer (Analytic Jena Specord 205). Measurements were recorded as μmol gallic acid equivalents (GAE) per gram extract. All determinations were performed in triplicate.

### 3.5. Antioxidant Activity (FRAP Assay)

The antioxidant activity of obtained extracts was measured using the ferric reducing antioxidant power (FRAP) assay according to Benzie and Strain [50]. Ferric to ferrous ion reduction at low pH (3.6 in acetate buffer) produces a colored ferrous-tripyridyltriazine complex. FRAP values are obtained by reading the absorbance change at 595 nm, which are linear over a wide concentration range [50]. The FRAP values were expressed as μmol Fe^2+^ equivalents per gram extract. All determinations were performed in triplicate.

### 3.6. Incubation and Direct Plating

The estimate of BHT and effect of natural extracts on seed fungal load was done with the direct plating method, according to standard protocol recommended by the International Commission on Food Mycology [51]. We used a selective medium for fungus, dichloran rose bengal chloramphenicol (DRBC) [52] and dichloran 18% glycerol (DG18) agar to detect the xerophilic *Aspergillus* and *Penicillium* which are capable to grow above 0,77 a_w_ from wheat flour and cereals [53]. For both medium all determinations were performed in triplicate. The Petri dishes were incubated at 25 ± 2 °C, with a frequency of 14 h darkness and 10 hours of diffuse light and observations on the growth of the fungus was made in 3 days binocular. The results were used to estimate the seeds contamination index (SCI) according to Doolotkeldieva [54], using the Formula (1):

(1) $$\text{SCI }(\%)=\frac{\text{number of contaminated seeds}}{\text{total number of seeds}}\times 100$$

The fungal colonies macroscopic identified as *Aspergillus*, *Penicillium* and *Fusarium* were sub cultured in MEA (malt extract 2%, peptone 0.1%, glucose 2%, agar 2%), PDA (potatoes infusion dry 20%, dextrose 2%, agar 1.5%) for taxonomic identification proposed by Pitt et Hochking [55].

For *Aspergillus flavus* and *parasiticus* confirmation AFPA medium (peptone 1%, yeast extract 2%, ferric ammonium citrate 0.05%, chloramphenicol 0.01%, agar 1.5% and dichloran 0.2% in ethanol) was used. The isolation frequency (Fr) of genera and relative density (RD) of OTA producer’s species were calculated according to Gonzalez [46] using the Formulas (2) and (3):

(2) $$\text{Fr }(\%)=\frac{\text{number of samples with a fungal genus}}{\text{total number of samples}}\times 100$$

(3) $$\text{RD }(\%)=\frac{\text{number of isolates of a species}}{\text{total number of fungi isolated}}\times 100$$

### 3.7. Mycotoxins Analysis

The method used in this study was enzyme-linked immunosorbent assay (ELISA). The ground samples (5 g) were extracted with 12.5 mL of methanol:water 70:30 (v/v) and shaken in a Waring blender at high speed for 20 min. The extract was filtered through a Whatman (Maidstone, UK) filter paper (No. 1). A 1-ml filtrate was diluted 1:1 with distilled water. Standard solutions and prepared samples (50 μL) were mixed with 50 μL of enzyme conjugate in individual dilution wells. Antibody solution (50 μL) was added and mixed gently by shaking the plate manually and incubated for 10 min at room temperature. Wells were washed three times with 250 μL distilled water. Substrate (100 μL) was added to each well and incubated for 5 min at room temperature. Following the addition of stop solution (100 μL) to each well, the intensity of the resulting yellow color was measured at a wavelength of 450 nm using an ELISA 96-well plate reader (PR-1100, Bio-Rad Laboratories, USA). The log-logit sheets supplied with the kits were used to generate a standard curve and to calculate the OTA content in the samples. All determinations were performed in triplicate. Prior to analysis of the samples, the ELISA method was validated to ensure data quality. Method validation was carried out by determination of recovery, standard deviations (SD), repeatability (RSDr) and reproducibility (RSDR), the minimum limit of detection (LOD) and limit of quantification (LOQ). In the present study, the average recoveries with their SD were 75.93 ± 0.2480, RSDr was 0.03978%, RSDR was 0.107%, LOD was 2.214 ppb and LOQ was 4.0383 ppb. These performance characteristics were in agreement with the limit accepted by the Commission’s Regulation for official method of OTA analysis [6].

### 3.8. Statistical Analysis

Results are presented as means ± standard deviation (SD) of triplicate measurements. The data in the present work were subjected to analysis of variance (ANOVA one-way) and the least significant difference test, in order to compare the mean values of the investigated parameters. Computations Tukey *post-hoc* means comparisons and Levene’s test for equal variance were also included.

Statistically significant differences are marked (*) and indicate a *p* value < 0.05. Statistically highly significant differences are marked (**) and indicate a *p* value <0.01. Statistically extremely significant differences are marked (***) and indicate a *p* value < 0.001. Statistical processing of data was performed using the Statistical Analysis System—SAS (software version 8.1; SAS Institute, Inc., Cary, NC, USA, 2000).

## 4. Conclusions

It could be concluded from the results of this study that the total fungal populations and OTA production were significantly reduced by the presence of natural extracts. The seed contamination index decreased in the presence of antioxidants. Also, the present study confirms the inhibitory effect of natural extracts obtained from wine industry by-products on *Aspergillus ochraceus* and *Penicillium verrucosum* growth during the storage period. Based on the observations carried out on the capacity of natural extracts to control growth and fungus development, *i.e.*, ochratoxin A synthesis, we can say that the best results were obtained on treatment with GPE. Due to their remarkable antioxidant properties, the extracts obtained from wine industry by-products can be recommended as natural additives in the food industry. GPE and GSE are able to provide fungicidal and fungistatic protection and control of OTA accumulation in wheat grain at least at a similar level to BHT.

The efficacy of these extracts for prevention or control of fungal development and OTA accumulation in wheat grain, makes them highly recommendable as additives in antifungal treatments applied to cereals destined for human consumption or feed. Furthermore, being processed from a low-cost material resulting from by-products from the wine industry, makes these extracts a valuable alternative to conventional methods used for control of OTA production in stored cereals.

## Figures and Tables

**Table 1:** Antioxidant properties of natural extracts and BHT.

Sample	FRAP Value (μmol Fe^2+^/g)	Total Phenolics (μmol gallic acid/g)
GSE	1042.38 ± 38.69	795.83 ± 32.18
GPE	804.17 ± 29.54	561.28 ± 26.41
BHT	1328.14 ± 56.71	-

**Table 2:** Changes in OTA content of wheat grain during storage showing effect of treatment with natural extracts and BHT.

Sample	OTA (ppb)				

	Period (days)				

	0	7	14	21	28
Control	12.93 ± 0.17	13.15 ± 0.35 ns	13.32 ± 0.26 ns	13.67 ± 0.25 *	14.12 ± 0.32 ***
500 ppm GSE	12.93 ± 0.17	13.41 ± 0.29 ns	12.28 ± 0.34 *	11.07 ± 0.32 ***	10.28 ± 0.47 ***
1000 ppm GSE	12.93 ± 0.17	12.08 ± 0.29 *	11.29 ± 0.36 *	10.90 ± 0.34 **	10.38 ± 0.37 ***
2500 ppm GSE	12.93 ± 0.17	11.68±0.46 *	10.62 ± 0.49 *	11.09 ± 0.39 *	9.42 ± 0.41 ***
500 ppm GPE	12.93±0.17	12.78 ± 0.37 ns	11.68 ± 0.35 **	10.83 ± 0.44 **	8.89 ± 0.48 ***
1000 ppm GPE	12.93±0.17	14.49 ± 0.43 *	11.21 ± 0.50 **	10.98 ± 0.54 **	9.00 ± 0.44 ***
2500 ppm GPE	12.93±0.17	12.27 ± 0.57 ns	11.96 ± 0.52 ns	10.45 ± 0.34 ***	9.01 ± 0.32 ***
500 ppm BHT	12.93±0.17	11.98 ± 0.33 *	10.79 ± 0.36 **	10.33 ± 0.45 **	10.17 ± 0.37 **
1000 ppm BHT	12.93±0.17	10.48 ± 0.38 *	9.55 ± 0.46 **	9.43 ± 0.32 **	9.32 ± 0.27 **
2500 ppm BHT	12.93±0.17	15.09 ± 0.43 *	10.74 ± 0.45 **	9.86 ± 0.48 **	9.12 ± 0.33 ***

Data are shown as means, relative to control (C) response recorded in the wheat grain in initial time (0). Statistical differences are indicated as:

ns non-significant (*P* > 0.1);

* significant (*P* < 0.05);

** highly significant (*P* < 0.01);

*** extremely significant (*P* < 0.001).

**Table 3:** The changes of SCI during storage showing effect of treatment with natural extracts and BHT.

Sample	SCI (%)				

	Period (days)				

	0	7	14	21	28
Control	83.33 ± 5.77	80.00 ± 0.00 ns	83.33 ± 5.77 ns	76.67 ± 5.77 ns	76.67 ± 5.77 ns
500 ppm GSE	83.33 ± 5.77	80.00 ± 0.00 ns	50.00 ± 0.00 **	36.67 ± 5.77 ***	43.33 ± 5.77 **
1000 ppm GSE	83.33 ± 5.77	90.00 ± 0.00 ns	56.67 ± 5.77 **	33.33 ± 5.77 ***	33.33 ± 5.77 ***
2500 ppm GSE	83.33 ± 5.77	80.00 ± 0.00 ns	56.67 ± 5.77 **	33.33 ± 5.77 ***	33.33 ± 5.77 ***
500 ppm GPE	83.33 ± 5.77	90.00 ± 0.00 ns	43.33 ± 5.77 **	40.00 ± 0.00 **	43.33 ± 5.77 **
1000 ppm GPE	83.33 ± 5.77	86.66 ± 5.77 ns	33.33 ± 5.77 **	33.33 ± 5.77 **	40.00 ± 0.00 **
2500 ppm GPE	83.33 ± 5.77	96.66 ± 5.77 ns	73.33 ± 5.77 *	40.00 ± 0.00 ***	53.33 ± 5.77 ***
500 ppm BHT	83.33 ± 5.77	80.00 ± 10.00 ns	56.67 ± 5.77 **	56.67 ± 5.77 **	60.00 ± 0.00 **
1000 ppm BHT	83.33 ± 5.77	76.67 ± 5.77 ns	96.67 ± 5.77 *	60.00 ± 0.00 ***	53.33 ± 5.77 ***
2500 ppm BHT	83.33 ± 5.77	80.00 ± 0.00 ns	53.33 ± 5.77 **	50.00 ± 0.00 **	46.67 ± 5.77 **

Data are shown as means, relative to control (C) response recorded in the wheat grain in initial time (0). Statistical differences are indicated as:

ns non-significant (*P* > 0.1);

* significant (*P* < 0.05);

** highly significant (*P* < 0.01);

*** extremely significant (*P* < 0.001).

## Abbreviations

OTA: ochratoxin A
BHA: butylated hydroxianisole
BHT: butylated hydroxytoluene
PP: propyl paraben
GSE: grape seeds extract
GPE: grape pomace extract
C: control (untreated sample)
FRAP: ferric reducing antioxidant power
SCI: seed contamination index
Fr: isolation frequency of genera
RD: relative density
AFPA: *Aspergillus flavus* and *parasiticus* agar medium
DRBC: dichloran rose bengal chloramphenicol agar medium
DG18: dichloran 18% glycerol agar medium
RSDr: repeatability
RSDR: reproducibility
LOD: minimum limit of detection
LOQ: limit of quantification.
